# Complete Genome Sequences of *Bordetella flabilis*, *Bordetella bronchialis*, and “*Bordetella pseudohinzii*”

**DOI:** 10.1128/genomeA.01132-16

**Published:** 2016-10-13

**Authors:** Theodore Spilker, Rebecca Darrah, John J. LiPuma

**Affiliations:** a Department of Pediatrics and Communicable Diseases, University of Michigan Medical School, Ann Arbor, Michigan, USA; b Department of Genetics and Genome Sciences and Frances Payne Bolton School of Nursing, Case Western Reserve University, Cleveland, Ohio, USA

## Abstract

We report here the complete genome sequences of *Bordetella flabilis* and *Bordetella bronchialis* recovered from cultures of individuals with cystic fibrosis (CF), and “*Bordetella pseudohinzii*” recovered from a CF mouse model.

## GENOME ANNOUNCEMENT

Individuals with cystic fibrosis (CF) are susceptible to infection of the respiratory tract with *Achromobacter* and *Bordetella* species (1–4). Correct identification of these phylogenetically closely related species may have prognostic implications and impact treatment. A recently published multilocus sequence typing scheme for *Achromobacter* revealed several novel species (5), many of which now have been taxonomically described and validly named (6–8). Sequence analysis of a fragment of *nrdA* was used to differentiate *Achromobacter* and *Bordetella* species (2–4) and identified a number of putative novel *Bordetella* species (3). Among these are the recently named *Bordetella bronchialis* and *Bordetella flabilis* (9). Another *Bordetella* genogroup, for which the name “*Bordetella pseudohinzii*” has been proposed, carries a clustered regularly interspaced short palindromic repeat (CRISPR)-associated protein 9 (Cas9) system (10). To gain further insight into the genetics of these species, we performed whole-genome sequence analysis of four strains: *B. bronchialis* AU3182, recovered from a CF patient in 2001; *B. bronchialis* AU17676, recovered from a CF patient in 2009; *B. flabilis* AU10664, recovered from a CF patient in 2006; and *B. pseudohinzii* HI4681, recovered in 2012 from bronchoalveolar lavage fluid of a C57BL/6 mouse homozygous for mutant *cftr*.

Bacteria were grown in Mueller-Hinton broth overnight at 37°C in an orbital shaker. Five milliliters of bacterial culture was pelleted and resuspended in 1 ml of 1× Tris-EDTA (TE) buffer to a concentration of ~10^8^ CFU/ml. Genomic DNA was extracted from 350 µl of the suspension using the MagNA Pure compact nucleic acid isolation kit (Roche), according to the manufacturer’s instructions. Genomic DNA libraries were prepared using an Illumina TruSeq DNA library kit and sequenced on an Illumina HiSeq 4000 paired-end flow cell (2 × 150-bp read length, V4 chemistry). Output files containing the fastq reads were checked and edited using Trimmomatic-0.33 (11). Read correction and assembly of draft genomes were carried out using SPAdes-3.7.1 (12). Genomes were annotated using NCBI’s whole-genome shotgun (WGS) submission portal containing the automated Prokaryotic Genomic Annotation Pipeline (PGAP) option.

The contigs of each draft genome were aligned to several complete *Bordetella* genomes available at NCBI, including, but not limited to, *Bordetella avium* strain 197N, *Bordetella bronchiseptica* strain 253, *Bordetella hinzii* strain H568, *Bordetella trematum* strain H044680328, and “*Bordetella* species” strain N, with Mauve version 2.4.0 (13). The reference-sorted draft genomes were manually gap filled by identifying short segments (20 to 25 bp) on the ends of two contiguous pieces that matched to both ends of a single contig of the draft genome not already included by Mauve in the alignment to the reference. These matches were verified by obtaining the longest possible perfect match on both sets of ends, checked with BLASTN for continuity, confirmed with BLASTX when possible, and checked for the appropriateness of gap distance against the reference strain. The genomes were annotated using NCBI’s whole-genome shotgun submission portal containing the automated Prokaryotic Genomic Annotation Pipeline (PGAP) option. The complete genomes, not including plasmids, ranged from 4,490,371 bp to 5,966,919 bp in length and contained 4,130 to 5,194 coding sequences (CDS) encoding proteins.

### Accession number(s).

This genome project PRJNA318508 has been deposited in GenBank under the accession numbers CP016170 to CP016173, CP016440, and CP016441.
